# P2X4R Overexpression Upregulates Interleukin-6 and Exacerbates 6-OHDA-Induced Dopaminergic Degeneration in a Rat Model of PD

**DOI:** 10.3389/fnagi.2020.580068

**Published:** 2020-11-04

**Authors:** Jiangnan Ma, Jinzhao Gao, Mengyue Niu, Xiaona Zhang, Jing Wang, Anmu Xie

**Affiliations:** ^1^Department of Neurology, Affiliated Hospital of Qingdao University, Qingdao, China; ^2^Department of Neurology, Ruijin Hospital of Shanghai Jiaotong University, Shanghai, China

**Keywords:** Parkinson’s disease, P2X4R, overexpression, IL-6, dopaminergic neuron

## Abstract

The pathogenesis of Parkinson’s disease (PD) remains elusive. Current thinking suggests that the activation of microglia and the subsequent release of inflammatory factors, including interleukin-6 (IL-6), are involved in the pathogenesis of PD. P2X4 receptor (P2X4R) is a member of the P2X superfamily of ion channels activated by ATP. To study the possible effect of the ATP-P2X4R signal axis on IL-6 in PD, lentivirus carrying the P2X4R-overexpression gene or empty vector was injected into the substantia nigra (SN) of rats, followed by treatment of 6-hydroxydopamine (6-OHDA) or saline 1 week later. The research found the relative expression of P2X4R in the 6-OHDA-induced PD rat models was notably higher than that in the normal. And P2X4R overexpression could upregulate the expression of IL-6, reduce the amount of dopaminergic (DA) neurons in the SN of PD rats, suggesting that P2X4R may mediate the production of IL-6 to damage DA neurons in the SN. Our data revealed the important role of P2X4R in modulating IL-6, which leads to neuroinflammation involved in PD pathogenesis.

## Introduction

PD is a common neurodegenerative disease, which has been recognized as presenting with a broad spectrum of the motor (including bradykinesia, resting tremor, and rigidity)and non-motor symptoms (including olfactory dysfunction, cognitive impairment, psychiatric symptoms, rapid eye movement sleep behavior disorder, and orthostatic hypotension), and varying disease progression (Weintraub et al., 2008; Ma et al., 2019; Tao et al., 2019; LeWitt et al., 2020; Zhang et al., 2020). A variety of non-motor symptoms lead to a decline in the quality of life and an increase in the economic healthcare burden (LeWitt et al., 2020). Based on the heterogeneous clinical presentations of PD, the future trend of biologic PD subtyping is finding consensus *via* clustering from multiple data sets and promoting its wide application in research and clinical practice. Identification of distinct subtypes would contribute to more focused studies of the etiology, pathophysiology, and disease-modifying treatment of PD (Qian and Huang, 2019).

PD is characterized by three basic pathological features: (a) Abnormalities in the DAergic system and its receptors in the basal ganglia, which are the pathological basis of motor symptoms in PD (Rangel-Barajas et al., 2015). (b) Non-enzymatic degenerative protein modifications (DPMs) and aggregation of α-synuclein (α-syn) into proteinaceous inclusions (Lewy bodies, LBs; Adav and Sze, 2020) and (c) Immune activation dysfunction (Rangel-Barajas et al., 2015; Diack et al., 2016; Tyson et al., 2016). Positron emission tomography (PET) scan showed significant hypometabolic activity in the bilateral putamen, caudate, anterior cingulate, left parietal lobe, and the prefrontal cortex in PD. Caudate metabolic activity and the prefrontal area both were negatively correlated with the H-Y stage, which were potentially applied as markers to evaluate the severity of PD (Chu et al., 2019). In terms of gross anatomical characteristics, the volumes of the bilateral amygdala and the left primary motor cortex were commonly reduced; while right entorhinal cortex atrophy may serve as a neuroanatomical biomarker in early, drug-naive PD with cognitive impairment (MCI) and help to discriminate MCI from non-demented PD (Jia et al., 2019). A sandwich enzyme immunoassay was applied to measure the tyrosine hydroxylase (TH) contents and activity in the nigrostriatal pathway in the control and PD group. The results showed both TH contents and activity decreased in the PD group as compared with those in the control group, and the decrease of TH activity in PD was attributed to the reduction of TH contents caused by neuron degeneration (Mogi et al., 1988).

Although the precise etiology of PD is still under exploration, genetic and environment factors (including pesticides, metals and solvents exposures, rural living, etc.), aging, autophagy, oxidative stress, renin-angiotensin system, together with changes in gut microbial composition and neuroimmunity are suggested to contribute to the neurodegeneration of DA neurons (Rangel-Barajas et al., 2015; Lai et al., 2018; Zhao et al., 2018; Adav and Sze, 2020; Zheng et al., 2020). Recently, the influence of dysregulated higher inflammatory response in the pathogenesis of central nervous system (CNS) diseases is attracting considerable attention (Hunot and Hirsch, 2003; Shen et al., 2019; Zhu et al., 2019; Yang et al., 2020). The abnormal inflammatory signaling modulates the activation of astrocytes and microglia, the release of inflammatory factors, ultimately the degeneration of DA neurons (Hunot and Hirsch, 2003; McGeer and McGeer, 2004; Frank-Cannon et al., 2009; Phani et al., 2012; Pradhan and Andreasson, 2013). Plasma antioxidants, such as the superoxide dismutase (SOD), are significantly reduced in PD, while inflammatory factors, such as high-sensitivity C-reactive protein (hsCRP), are increased (Jin et al., 2020), which suggested that oxidative stress and inflammation in the peripheral blood may be involved in the pathogenesis of PD (Adav and Sze, 2020; Yang et al., 2020). Lower SOD and higher hsCRP are important indicators to evaluate the severity of PD (Yang et al., 2020).

One of the main changes in aging is the dysregulation of the immune reactions, which leads to chronic systemic inflammation. Cytokines and chemokines are major culprits in the development of chronic inflammation and the immunosenescence process. The nuclear factor (NF)-κB exerts its role in maintaining immune responses *via* activation of pro-inflammatory cells and upregulation of the expression of pro-inflammatory mediators, such as CRP and IL-6, which has a close relationship to a various age-related chronic pathological state. Compared to the young, the circulating levels of inflammatory cytokines are typically higher in the elderly. During aging, the increase in immune cell infiltration leads to enhanced chronic inflammation (Chung et al., 2019). Through the study of the inflammatory signaling pathway, it is of considerable significance to provide insights into the pathogenesis and therapy of PD.

The release of adenosine 5’-triphosphate (ATP) is a widespread mechanism for intercellular communication (Montilla et al., 2020). In the presence of inflammation, extracellular ATP increases rapidly to near millimolar levels, which mediates the activation of pro-inflammatory pathways (Burnstock, 2016). Separate families of receptors for adenosine (P1 receptors), ATP, and adenosine diphosphate (ADP; P2 receptors) were put forward in 1978 (Fields and Burnstock, 2006). All neurons, astrocytes, microglia, and oligodendrocytes express P1 and P2 receptors (North, 2002; Burnstock, 2015; Montilla et al., 2020). P2 receptors are divided into metabotropic P2Y receptors (P2YRs) that are G-protein-coupled and ionotropic P2X receptors (P2XRs) that are nucleotide-gated ion channels (Burnstock and Kennedy, 1985; Burnstock, 2004; Suurväli et al., 2017). Both the metabotropic and ionotropic receptors are located at presynaptic (axonal terminals) and postsynaptic (dendritic) sites, regulating either neurotransmitter release or the postsynaptic sensitivity to neurotransmitters (Koles et al., 2011; Burnstock, 2015). Purinergic signaling participates in normal physiological behaviors, such as learning and memory, sleep and arousal, locomotor activities, and cognition. P2X receptors are also involved in the pathological processes of the brain, including injury, inflammation, Alzheimer’s disease (AD), PD, multiple sclerosis (MS), depression, and anxiety (Burnstock, 2015). Seven distinct P2XR subtypes (P2X1-7R) and eight P2YR subtypes (P2Y1, 2, 4, 6, 11–14) have been cloned from mammals (North, 2002; Abbracchio et al., 2006; Burnstock, 2011; Koles et al., 2011). P2X4R is one of the most sensitive purinergic receptors, which was first cloned from the brain and typically expressed by neurons and microglia (Nörenberg and Illes, 2000; Fields and Burnstock, 2006; Burnstock, 2007; Paalme et al., 2019).

ATP can bind to specific purinergic receptors as an extracellular signaling molecule, and mediates diverse cellular processes such as trans-synaptic transmission, inflammation progress, macrophage activation, cell differentiation and proliferation, neuropathic pain (Ihara et al., 2005; Burnstock, 2015; Suurväli et al., 2017; Tóth et al., 2019). Extracellular ATP induces Ca-mediated activation of microglia *via* binding to P2X/P2YRs. After activation, microglia showed enhanced phagocytic activity, activation of the inflammasome, and release of cytokines (so-called M1/M2 polarization; Gilbert et al., 2016).

Microglia are primary immune cells in the brain and major for the initiation and resolution of inflammation caused by pathogen or tissue damage (Schiess et al., 2010; George et al., 2019). Microglial activation signifies a primary inflammatory state, causes secondary leukocyte invasion which amplifies inflammation, and strongly suggests the pathological and repair process of nervous system diseases, such as PD, AD, chronic inflammatory, and neuropathic pains (Guo et al., 2005; Ladeby et al., 2005; Phani et al., 2012; Jurga et al., 2017; Lalisse et al., 2018; Long et al., 2018). Moreover, astrocytes and oligodendrocytes also participate in the inflammatory process by producing or responding to pro-inflammatory mediators (Chung et al., 2019). Previous research has shown that astrocyte reactivity was detected in the substantia nigra (SN) pars compacta of PD. At the time of PD onset, α-syn accumulated in astrocytes, then phagocytic microglia are recruited to attack certain neurons in the restricted brain region, leading to the clinical symptoms of PD (Li et al., 2019). Autopsy study suggested that there were abundant activated microglia and lymphocytic infiltration of CD4+ and CD8+ T cells in the SN of PD, the number of which was proportional to the degree of neurodegeneration (Hirsch et al., 1998). These findings suggested that immune cells recruitment and local inflammatory reactions serve as significant factors causing neurodegeneration of the nigrostriatal pathway (Zheng et al., 2020). P2X4R has a great effect on chemotaxis and motility of microglia (Montilla et al., 2020). P2X4R upregulation has been proved in multiple models associated with microglial activation, which is common in most neurodegenerative diseases related to inflammation (Montilla et al., 2020).

IL-6 is one of the most important cytokines in the inflammatory process. Increased circulating IL-6 is a hazard factor for multiple diseases (Shen et al., 2019). The high levels of IL-6 are associated with poor physical performance (slower gait velocity and muscle weakness), depression, and cognitive impairment (Banerjee et al., 2017; Chakrabarti et al., 2017; Ma and Chan, 2020). IL-6 and the IL-6 receptors promote chronic inflammation in the CNS and lead to the progression of PD, AD, stroke, and other nervous system diseases (Banerjee et al., 2017; Shen et al., 2019; Ma and Chan, 2020). IL-6 can modulate the blood-brain barrier (BBB) properties. BBB disruption might lead to more inflammatory cells infiltration. The level of IL-6 at the early stage of stroke is negatively correlated with the prognosis of neurological function and might be considered as a key biomarker to predict the prognosis (Shen et al., 2019). The inflammatory index score based on IL-6 was reported to best describe age-related chronic inflammation (Ma and Chan, 2020).

The autopsy revealed the amounts of different inflammatory factors including Tumor Necrosis Factor-α (TNF-α), IL-1β, IL-2, IL-4, IL-6 were notably higher both in the striatum and in cerebrospinal fluid of PD than the normal (Mogi et al., 1995; 1996a,b; Nagatsu et al., 2000). Mogi et al. (1994) found that the immunoreactivity of IL-6 in the SN of PD was prominently higher than that in the control group, indicating a distinct association between IL-6 and PD. In the same study, the expression level of IL-6 showed a significant increase in the striatum of PD patients *via* ELISA assay. However, no such changes were noticed in the cerebral cortex (Mogi et al., 1994). These observations suggest that the increase in cytokines may present not only as a compensatory response but as a primary initiating trigger for the neurodegeneration (Mogi et al., 1994, 1996b). After chronic constriction injury (CCI), repeated administration of P2X4R antagonist lowers the level of IL-6, implying P2X4R may modulate the activation of neuroglia and the release of IL-6 (Jurga et al., 2017).

6-OHDA, a neurotoxic synthetic organic compound, causes selective DA neuron loss and associated motor deficits in rodents (Becker et al., 2018; Tronci and Francardo, 2018). To further understand the correlation between the inflammatory response and the pathogenesis of PD, we constructed 6-OHDA-induced PD rat models, and detected the relative expression of P2X4R and IL-6, and the number of TH positive DA neurons in the SN of rats, and observed the changing trend of the above results after P2X4R overexpression. The objective of the experiment is to explore the role of P2X4R and inflammatory response in the pathogenesis of PD.

## Materials and Methods

### Animals

Experiments were conducted on the SPF male Wistar rats (age, 3–4 months; weight, 200–250 g) purchased from the Qingdao experimental animal center. Every four rats were fed in a clean cage lined with sawdust, under a 12 h light:12 h dark cycle (lights on at 06:00 a.m.), 55 ± 10% relative humidity, and 22.0 ± 2.0°C. Rats had unlimited access to standard chow and water. All the experimental procedures were carried out according to the National Institutes of Health Guide for the Care and Use of Laboratory Animals, and the protocols were approved by the Ethics Committee of the Affiliated Hospital of Qingdao University.

All rats were randomly assigned to six groups with each group containing 25 rats: control group, 6-OHDA group, lentivirus vectors carrying the P2X4R-overexpression target gene (P2X4R-OE) group, vector carrying P2X4R-negative control (P2X4R-NC) group, P2X4R-OE + 6-OHDA group, and P2X4R-NC + 6-OHDA group.

### Equipment and Reagent

A frozen microtome (Leica, Germany), brain stereotaxic apparatus (Reward, China), fluorescence microscope (Carl Zeiss, Germany), electrophoresis apparatus and Transfer Machine (BioRad, USA), 10 μl micro-syringe (Stoelting, USA), P2X4R lentivirus (Genechem, China, Titer 2E + 9TU/ml), P2X4R antibody (Alamnelabs, Israel), IL-6 antibody (Abcam, UK), tyrosine hydroxylase (TH) antibody (Millipore, USA), goat-anti-rabbit IgG and goat-anti-mouse IgG (Life Technologies, USA), 6-hydroxydopamine (Sigma, USA), apomorphine (Sigma, USA), isoflurane, isopropanol, anhydrous ethanol (Fuyu Chemical, China).

### Animal Surgery

Isoflurane (3% induction, 1.5% maintenance) anesthetized rats were firmly fixed on a stereotaxic apparatus. After disinfection with iodine, the scalp was cut through the mid-sagittal position to expose the bregma. According to the stereotaxic map of the rat brain, the coordinates relative to the bregma of left SN were as follows: AP – 5.0 mm, L 2.1 mm, and D 7.7 mm. Two microliter of either control virus or lentivirus carrying the target gene were injected with micro-syringe into the left SN of rats along the drilled microwell of the skull. One week later, 2 μl of normal saline (only 0.02% ascorbic acid) or 6-OHDA solution (8 μg/2 μl) was injected into the left SN of rats in the same manner.

### Treatment Groups

(1) Control group: normal saline was injected into the left SN. One week later, the animals were re-injected with saline.
(2) 6-OHDA group: 6-OHDA was injected into the left SN. One week later, the animals were re-injected with saline.
(3) P2X4R-OE group: lentivirus carrying the P2X4R-overexpression target gene was injected into the left SN. One week later, the animals were re-injected with saline.
(4) P2X4R-NC group: lentivirus carrying no load RNA was injected into the SN. One week later, the animals were re-injected with saline.
(5) P2X4R-OE + 6-OHDA group: lentivirus carrying the P2X4R-overexpression target gene was injected into the left SN. One week later, the animals were re-injected with 6-OHDA.
(6) P2X4R-NC + 6-OHDA group: lentivirus carrying no load RNA was injected into the left SN. One week later, the animals were re-injected with 6-OHDA.

### Apomorphine-Induced Rotation Test

**Figure 1:**
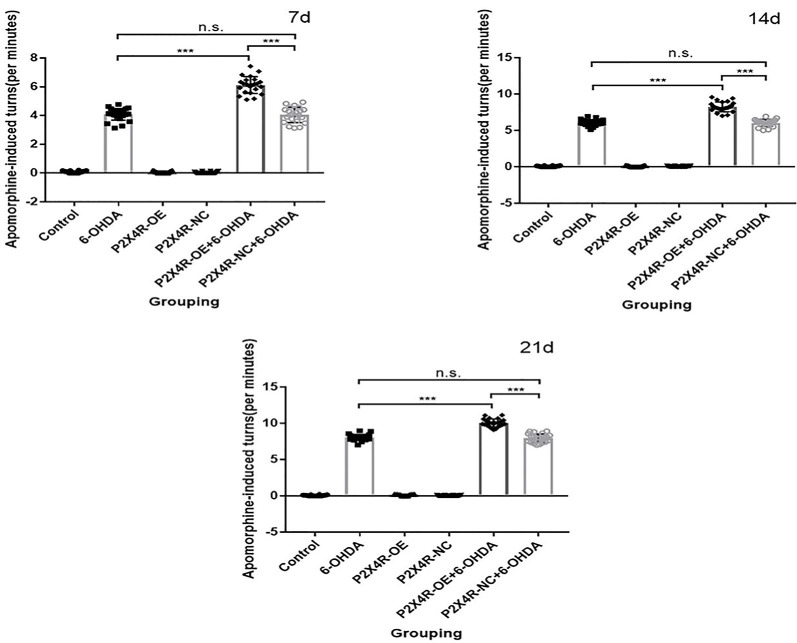
Apomorphine-induced rotation tests at 7, 14, and 21 Days. ***p < 0.001. n.s.: there is no statistical difference between the two groups.

All rats were injected with apomorphine (0.5 mg/kg, subcutaneously) at 7, 14, and 21 days after the 6-OHDA lesion. Count the number of rotations of each rat for 30 min. Further immunofluorescence and immunoblotting were performed only on those showed more than 7 turns/min of the whole body to the right in the third rotation test. There was no significant difference between the 6-OHDA group and the P2X4R-NC + 6-OHDA group (*P* > 0.05). P2X4R overexpression significantly increased the contralateral asymmetric rotation of the 6-OHDA injection site compared with the PD group (*P* < 0.001; Table 1, Figure 1), which suggested that overexpression of P2X4R aggravated the motor deficits of PD rat models. After excluding the dead and failing models, 22 rats were left in the control, 6-OHDA, and P2X4R-OE group, 20 rats were left in P2X4R-NC, and P2X4R-NC + 6-OHDA group, and 21 rats were left in the P2X4R-OE + 6-OHDA group.

**Table 1:** Rotation test results of rats in six groups (turns/min).

	Control	6-OHDA	P2X4R-OE	P2X4R-NC	P2X4R-OE + 6-OHDA	P2X4R-NC + 66-OHDA	*F*	*P*
*n*	22	24	23	22	23	22		
7 d	0 ± 0.10	4 ± 0.41	0 ± 0.10	0 ± 0.05	6 ± 0.59	4 ± 0.53	1,193.918	<0.001
14 d	0 ± 0.06	6 ± 0.41	0 ± 0.08	0 ± 0.10	8 ± 0.68	6 ± 0.48	2,202.965	<0.001
21 d	0 ± 0.07	8 ± 0.42	0 ± 0.10	0 ± 0.05	10 ± 0.55	8 ± 0.58	3,720.398	<0.001

*6-OHDA, 6-hydroxydopamine; P2X4R-OE, P2X4 receptor-overexpression; P2X4R-NC, P2X4 receptor-negative control; 7 d, 7 days; 14 d, 14 days; 21 d, 21 days*.

### Western Blot and Quantitative Analysis

After behavioral tests, 10 rats in each group were randomly selected for western blot, anesthetized with isoflurane, and decapitated for brain extraction. The left ventral midbrain was rapidly taken out and placed in EP tubes. Two-hundred microliter cell lysates with 2 μl protease inhibitor and 20 μl phosphatase inhibitor were added into each EP tube. After 30 min of incubation in ice, samples were centrifuged at 12,000 r/min for 15 min at 4°C, and the supernatant was taken. Protein concentrations in the SN were measured by the BCA assay. The target protein was separated by SDS-PAGE gel electrophoresis. The protein sample (20 μg per well) was added to each well at 80V constant current for 30 min, then the constant voltage was adjusted to 120V for 1 h. After transferring the separated protein to the PVDF membrane, the membrane was blocked with 0.05% skim milk at room temperature for 2 h, then incubated with primary antibodies overnight at 4°: P2X4R (1:200), β-actin (1:2,000), IL-6 (1:2,000). The next day, the membrane was incubated with HRP conjugated appropriate secondary antibodies (goat anti-mouse IgG, 1:10,000) for 1 h. Add chemiluminescence (ECL) reagents to visualize the protein bands. The density of each protein band was analyzed by Image-J software.

### Immunofluorescence and Quantitative Analysis

After behavioral tests, 10 rats in each group were randomly selected for immunofluorescence and anesthetized with isoflurane. The rats were intracardiac perfused with the normal saline and followed by 4% polyformaldehyde. The brain tissue was fixed with 4% polyformaldehyde overnight at 4°C, then incubated with 20% sucrose solution, followed by 30% sucrose solution to dehydrate successively. The brain tissue was cut into 20 μm thickness coronal slices at −20°C. The frozen sections were collected with a fine needle and placed in the 6-microwell plate containing 0.01 mol/l PBS in order, and incubated with TH antibody (1:2,000) overnight at 4°C, followed by goat-anti-rabbit IgG (1:500) in the dark for 2 h at room temperature. After rinsed with PBST three times, the sections were air-dried under dark conditions. After covering with cover glass with 70% glycerophosphate buffer solution, the sections were stored at 4°C. The successful transfection of lentivirus in the SN was observed with a confocal fluorescence microscope and photographed under 100× magnification. The TH positive neurons were counted in a fixed sequence under 400× magnification. Image-J software was applied to count the number of TH positive neurons in parallel brain sections.

All analyses were conducted using SPSS and Graphpad prism 7. All data are presented as the mean ± SD. The results accorded with the normal distribution and were analyzed by one-way analysis of variance (ANOVA) followed by LSD *post hoc* comparisons. Statistical significance was taken at *p* < 0.05.

## Results

### The Western Blot Analysis of the Protein Levels

#### Detection of P2X4R Expression in the SN by Western Blot

**Figure 2:**
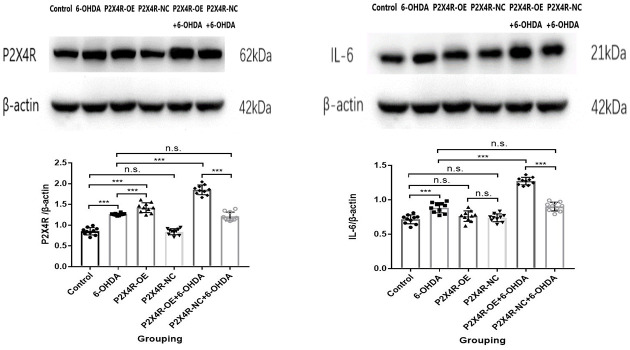
The representative bands and western blot analysis of P2X4R and IL-6 in the substantia nigra (SN; *n* = 10). ***P < 0.001; n.s.: there is no statistical difference between the two groups.

Compared to the control group, the expression of P2X4R in the 6-OHDA group and P2X4R-OE group was significantly increased (*P* < 0.001). The expression of P2X4R in the control group and the P2X4R-NC group had no significant difference (*P* > 0.05). Compared with the P2X4R-NC + 6-OHDA group, the expression of P2X4R protein in the P2X4R-OE + 6-OHDA group was significantly increased (*P* < 0.001). There was no significant difference in the expression of P2X4R between the 6-OHDA group and P2X4R-NC + 6-OHDA group (*P* > 0.05); compared with the 6-OHDA group, the expression of P2X4R in the P2X4R-OE + 6-OHDA group was significantly higher (*P* < 0.001; Table 2, Figure 2) Here comes the conclusion that the lentivirus carrying P2X4R-overexpression target gene is stably expressed in the SN of rats. The increased expression of P2X4R in PD rats induced by 6-OHDA suggests that P2X4R is involved in the pathogenesis and development of PD.

**Table 2:** Expression of P2X4R and IL-6 in the substantia nigra (SN) of rats in six groups.

	Control	6-OHDA	P2X4R-OE	P2X4R-NC	P2X4R-OE	P2X4R-NC	*F*	*P*
					+ 6-OHDA	+ 66-OHDA		
*n*	10	10	10	10	10	10		
P2X4R	0.8537 ± 0.0887	1.2620 ± 0.0260	1.4223 ± 0.1174	0.8425 ± 0.0737	1.8509 ± 0.1162	1.2095 ± 0.1056	163.647	<0.001
IL-6	0.7164 ± 0.0650	0.8855 ± 0.0734	0.7633 ± 0.0766	0.7390 ± 0.0572	1.2726 ± 0.0542	0.9020 ± 0.0638	100.051	<0.001

*6-OHDA, 6-hydroxydopamine; P2X4R-OE, P2X4 receptor-overexpression; P2X4R-NC, P2X4 receptor-negative control; IL-6, interleukin-6*.

#### Detection of IL-6 Expression in the SN by Western Blot

Compared to the control group, the expression of IL-6 in the SN of the 6-OHDA group was significantly increased (*P* < 0.001). There was no significant difference between the P2X4R-NC group and the P2X4R-OE group (*P* > 0.05). The expression of IL-6 in the SN of the P2X4R-OE + 6-OHDA group was significantly higher than that of the P2X4R-NC + 6-OHDA group (*P* < 0.001). Compared with the 6-OHDA group, the expression of IL-6 of the P2X4R-OE + 6-OHDA group was significantly higher (*P* < 0.001). There was no significant difference in IL-6 expression between the 6-OHDA group and the P2X4R-NC + 6-OHDA group (*P* > 0.05; Table 2, Figure 2). These data suggest that overexpression of P2X4R may further increase the amount of IL-6 in 6-OHDA-induced PD rats. However, overexpression of P2X4R does not cause a significant increase in IL-6 in normal rats.

### Overexpression of P2X4R Increases 6-OHDA-Induced Dopaminergic Neurodegeneration in the SN

**Figure 3:**
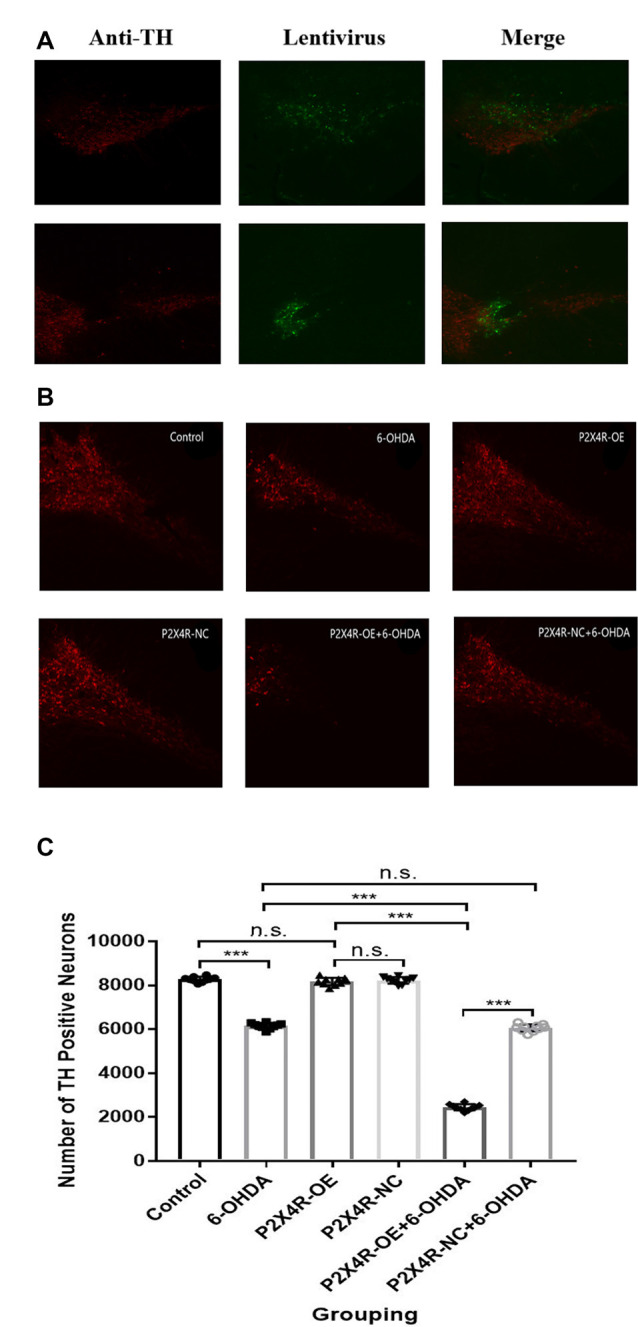
**(A)** Transfection of recombinant lentivirus in tyrosine hydroxylase (TH) positive DA neurons (x100). **(B)** Rrepresentative immunofluorescence pictures of six groups (x100). **(C)** Quantification of TH positive DA neurons (*n* = 10). ***P < 0.001; n.s.: there is no statistical difference between the two groups.

Compared to the control group, the number of TH positive DA neurons of the 6-OHDA group and the P2X4R-NC + 6-OHDA group decreased significantly (*P* < 0.001); compared to the P2X4R-NC + 6-OHDA group, the number of TH positive DA neurons of P2X4R-OE + 6-OHDA group decreased significantly (*P* < 0.001). There was no significant difference in TH positive DA neurons between the control group, the P2X4R-NC group, and the P2X4R-OE group (*P* > 0.05; Table 3, Figure 3). The number of DA neurons in the 6-OHDA-induced PD rats is significantly reduced, while the overexpression of P2X4R can further enhance the toxic effect on neurons.

**Table 3:** The number of tyrosine hydroxylase (TH) positive DA neurons in the SN of rats.

	Control	6-OHDA	P2X4R-OE	P2X4R-NC	P2X4R-OE	P2X4R-NC	*F*	*P*
					+ 6-OHDA	+ 66-OHDA		
*n*	10	10	10	10	10	10
DA neurons	8,276 ± 120.24	6,154 ± 138.45	8,173 ± 175.49	8,230 ± 151.66	2,452 ± 142.40	6,061 ± 158.39	2,315.953	<0.001

*6-OHDA, 6-hydroxydopamine; P2X4R-OE, P2X4 receptor-overexpression; P2X4R-NC, P2X4 receptor-negative control; DA, dopaminergic*.

## Discussion

With the advent of an aging population, the prevalence of PD gradually increases (Xie et al., 2019). With the development of the disease process, a large proportion of PD patients are suffering from both increasing clinical symptoms and worsening of quality of life (Sun et al., 2018). Diverse non-motor symptoms may play a more significant role than motor symptoms in PD patients’ quality of life (Sun et al., 2018). Although PD has been deeply researched, the precise pathogenesis is still elusive, and consequently, treatment is mainly symptomatic rather than preventive. Long-term use of DOPA preparations will lead to severe complications such as dyskinesia and fluctuation of symptoms. As a result, it is imperative to seek effective therapeutic approaches by studies on the pathogenesis of PD, including modulation of neuronal-vascular pathways, neuroinflammation, and neuronal autophagy, etc. (Rangel-Barajas et al., 2015; Zou et al., 2017; Weng et al., 2018; Zhang et al., 2018; Liu et al., 2019; Tian et al., 2019). For example, Cystatin C (CYS C) participates in neurovascular units activity by interacting with the vascular endothelial growth factors (VEGFs) and autophagy pathways. CYS C is considered as a potential therapeutic target to PD by VEGF-mediated angiogenesis and enhanced neuronal autophagy (Zou et al., 2017; Weng et al., 2018). CYS C is also applied to monitor and evaluate the severity of PD, AD, and progressive supranuclear palsy (PSP; Weng et al., 2018). Human Urinary Kallidinogenase (HUK) acts as a potential neuroprotectant, functioning to increase the expression of VEGF and promote angiogenesis and neuronal regeneration in hypoxic-ischemic encephalopathy (HIE) models, which may be an alternative treatment to PD (Gao et al., 2018). Upregulation of autophagy may be considered as a new therapeutic approach *via* inhibiting the activation of caspase-1, simultaneously leading to weakness of neuroinflammation and accelerating the elimination of misfolded proteins (Lai et al., 2018). Sirtuin3 (SIRT3) causes autophagy to ameliorate rotenone-induced cell damage, which may be a promising drug target for PD (Zhang et al., 2018). Another drug that may have therapeutic effects on PD by enhancing autophagy, avoiding excessive neuronal death is dihydroartemisinin (DHA; Zhao et al., 2020). Deep brain stimulation (DBS) is a prospective treatment for PD, especially in patients with dementia, anxiety, and addiction (Tan et al., 2020). Novel behavioral treatment techniques may provide new directions for PD patients, such as Lee Silverman Voice Treatment for dysarthria (Yuan et al., 2020). From the current research, the inflammatory response is closely related to the occurrence and development of PD. The activation of P2X4R plays a significant role in the inflammatory response, but the exact relationship between P2X4R and inflammatory factors is not clear.

In this study, we constructed PD rat models *via* intracranial injection of 6-OHDA and investigated whether P2X4R overexpression can mediate the neuro degeneration and neuroinflammatory response. The results revealed that P2X4R overexpression significantly increased the contralateral asymmetric rotation of the 6-OHDA injection site compared with the PD group, which suggested that overexpression of P2X4R aggravates the behavioral abnormalities induced by 6-OHDA. Compared to the control group, the expression of P2X4R in the P2X4R-OE group increased significantly, which demonstrated that the P2X4R-overexpression target gene carried by lentivirus was stably expressed in the SN of rats. The relative expression of P2X4R in the PD group was significantly higher than that in the normal control group, and the number of TH positive DA neurons in the SN decreased inversely; the reduction of TH positive DA neurons in the SN was significantly more severe in the P2X4R-OE + 6-OHDA group compared to P2X4R-NC + 6-OHDA group. Collectively, our study suggests that P2X4R may play an essential role in the pathogenesis of PD by damaging DA neurons through some specific mechanisms. It has already been confirmed that presynaptic and postsynaptic DA markers were notably modified in the nigrostriatal system of P2X4R knockout (KO) mice, suggesting dopaminergic neurotransmission was affected. In the 6-OHDA-induced PD model, P2X4R KO mice developed an attenuated L-dopa-induced movement behavior, while ivermectin, a positive modulator of P2X4R, enhanced this behavior (Khoja et al., 2016). These data indicate that P2X4R take a significant role in maintaining dopamine homeostasis and associated behaviors.

Western blot was also applied to validate the expression of IL-6 in the SN. Compared with the control group, the relative expression of IL-6 in the SN of PD rats was increased; and the increase of IL-6 in the P2X4R-OE + 6-OHDA group was more significant, which was consistent with the change of P2X4R. The results showed that overexpression of P2X4R could upregulate the expression of IL-6 in the SN of PD, suggesting that P2X4R may mediate IL-6 to damage DA neurons. Mice lacking P2X4R showed reduced activation of the inflammasome and no response to inflammatory factors, which was consistent with our findings (Burnstock, 2016).

However, overexpression of P2X4R alone did not lead to a significant increase in IL-6 and a decrease of DA neurons. ATP releases from damaged cells or activated immune cells, glial cells, and endothelial cells (Burnstock, 2016). While an optional amount of extracellular ATP favors healthy CNS as neuromodulators, an unusually large amount of ATP released from damaged neurons activates neuroglia and triggers pathogenicity in many diseases, including ischemia, spinal cord injury, PD, AD, Huntington’s disease (HD), and multiple sclerosis (MS; Burnstock, 2007; Di Virgilio et al., 2009; de Rivero Vaccari et al., 2012; Pradhan and Andreasson, 2013; Verma et al., 2017; Di Virgilio and Sarti, 2018; Domercq et al., 2019). We speculated that in the case of ONLY P2X4R overexpression, excessive P2X4R activation, and subsequent release of inflammatory factors would not be caused due to the lack of ATP released by damaged neurons and activated glial cells. In conclusion, overexpression of P2X4R can further increase the amount of IL-6 and enhance the damage of DA neurons only when 6-OHDA lesion.

In cultured mouse primary microglia, activation of P2X7 receptor (P2X7R) by ATP induced the mRNA expression and release of IL-6, but not in P2X7(−/−) cells. Various selective P2X7R antagonists restrained the P2X7-dependent activation and release of IL-6 (Shieh et al., 2014). P2X4R has higher homology with P2X7R (about 40%) at the amino acid sequence level than other P2X receptor subtypes, and there is structural interaction between P2X4R and P2X7R (Ma et al., 2006; Qureshi et al., 2007). Co-expression of P2X4R with P2X7R enhances P2X7R-mediated inflammation through both promoting the release of cytokines and inhibiting autophagy (Kawano et al., 2012a; Sakaki et al., 2013). While P2X4R KO significantly inhibited cell death caused by ATP or P2X7 agonist (Kawano et al., 2012b).

Aging is the most significant risk factor of PD because of chronic neuroinflammation and oxidative stress microenvironment in the brain. Neuroimaging-driven brain age estimation has shown a significantly “older-appearing” brain of PD, and the aging of white matter is more serious than that of gray matter (Beheshti et al., 2020). Aging exaggerates inflammatory M1 microglia, impairs the DA neurons, and increases the expression of pro-inflammatory and pro-oxidative stress factors (Zhao et al., 2018). PET imaging studies *in vivo* showed the proliferation of microglia was in parallel with the appearance of pathological α-syn and neuronal death in PD patients (Zheng et al., 2020). Upon M1 polarization, microglia produce pro-inflammatory cytokines and neurotoxic molecules; while anti-inflammatory mediators are released when microglia shift to the M2 phenotype (Eggen et al., 2013).

We conclude that in PD, ATP binds to P2X4R on the surface of microglia, and then activates microglial downstream signaling pathways to produce cytokines and other interleukin factors, such as IL-6 (Meng et al., 2017). The released cytokines then bind to their respective receptors on the microglial surface to further accelerate the activation of microglia (Hide et al., 2000; Sanz and Di Virgilio, 2000; Long-Smith et al., 2010; Chertoff et al., 2011; McCoy et al., 2011; Verderio and Matteoli, 2011). The vicious cycle of inflammation activated by microglia may be involved in the occurrence and development of PD, epilepsy, and other neurological diseases (Inoue et al., 2007; Frank-Cannon et al., 2009; Koles et al., 2011; Trang et al., 2012; Alves et al., 2019). Therefore, suppression of inflammation of microglia and astrocytes plays an important role in inhibiting the death of neurons. For example, nuclear receptor related1 (Nurr1) is a key regulator of inhibiting the expression of inflammatory genes in microglia and astrocytes. Nurr1 is associated with the differentiation of DA neurons; while Nur77 plays a key role in inflammation and apoptosis. Nurr1 agonists/mimetics may serve as potential therapeutic targets for PD and other neurodegenerative disorders. Memantine exerts its neuroprotection by the regulation of the contra-directional coupling between Nur77 and Nurr1 (Wei et al., 2016; Jeon et al., 2020). Decreased serum trefoil factor 3 (TFF3)/cholinesterase (ChE) activity and increased homocysteine (Hcy) are associated with vascular function, inflammation, and oxidative stress, which may underlie the pathophysiological mechanisms of PD dementia (PDD) and vascular parkinsonism with dementia (VPD). Serum TFF3, ChE activity, and Hcy could be used to evaluate the severity of PDD and VPD (Zou et al., 2018). Infiltrating T cells are involved in propagating neurodegeneration. Intestinal microbial signals provide clues for the differentiation of gut regulatory T (Treg) cell subsets. Treg cells not only limit immune activation but also mediate the protection of the nigrostriatal system by inhibiting microglial inflammation and the cytotoxic activity of CD8+ T cells and NK cells against α-syn aggregation. Therefore, the regulation of intestinal microflora may exhibit an inhibitory effect on peripheral and CNS inflammation (Zheng et al., 2020). SOD is shown as a therapeutic target for preventing cognitive dysfunction development and reducing its severity in cerebral small vessel disease, which also has enlightenment for the treatment of PD with cognitive impairment (Zhu et al., 2019).

Treatment based on purinergic receptors may help to prevent excessive inflammation and promote the repair of neuroinflammatory disorders. P2X4R has emerged as a potential target for CNS diseases such as PD, epilepsy, ischemia, chronic pain, anxiety, and MS (Montilla et al., 2020). Inhibitory monoclonal antibodies binding the head domain of P2X4R, selective P2X4R antagonist, allosteric modulator, and inhibitor for P2X4R-mediated Ca-responses have therapeutic potential for anti-inflammatory and anti-nociceptive effects (Gilbert et al., 2016; Jacobson and Müller, 2016; Pasqualetto et al., 2018; Zabala et al., 2018; Ase et al., 2019; Hu et al., 2019; Werner et al., 2019; Williams et al., 2019). Based on the diverse functional roles of IL-6 in the CNS, different treatment strategies can be carried out. Therefore, the effective use of IL-6 antagonists, soluble IL-6 receptors provides new treatment strategies for neurodegenerative diseases (Inoue, 2006). Exercise training and increased physical activity have positive effects on many chronic diseases that consistent with the inflamm-aging paradigm, which serves as a new direction of anti-inflammatory treatment for PD (Flynn et al., 2019). Besides, inflammatory cells in peripheral blood also participate in the pathogenesis of PD. Cannabinoid receptor 2 (CB2) agonists, which decrease the recruitment of neutrophils and downregulate pro-inflammatory cytokine production, may limit inflammation and delay the progress of PD (Cioni et al., 2019; Hussain et al., 2019).

These findings provide a solid evidence base that lentivirus-mediated P2X4R overexpression modulates the upregulation of IL-6 in the SN of PD rats, which can damage DA neurons and affect the pathogenesis and development of PD. Our study provides a further theoretical basis for clarifying the etiology of PD.

## Data Availability Statement

All datasets presented in this study are included in the article.

## Ethics Statement

The animal study was reviewed and approved by the Ethics Committee of the Affiliated Hospital of Qingdao University.

## Author Contributions

AX and JW directed the design of the experimental scheme and the writing of the article. JM and JG were responsible for doing the experiment and writing the article. MN and XZ were responsible for revising the article. All authors contributed to the article and approved the submitted version.

## Conflict of Interest

The authors declare that the research was conducted in the absence of any commercial or financial relationships that could be construed as a potential conflict of interest.
